# The Retinal Renin-Angiotensin-Aldosterone System: Implications for Glaucoma

**DOI:** 10.3390/antiox11040610

**Published:** 2022-03-22

**Authors:** Kazuyuki Hirooka, Yoshiaki Kiuchi

**Affiliations:** Department of Ophthalmology and Visual Science, Hiroshima University, Hiroshima 734-8551, Japan; ykiuchi@hiroshima-u.ac.jp

**Keywords:** renin-angiotensin-aldosterone system, glaucoma, primary aldosteronism, oxidative stress

## Abstract

Aldosterone is one of the main effectors of the renin-angiotensin-aldosterone system (RAAS) along with having roles in hypertension, and cardiovascular and renal diseases. Recent evidence has also shown the presence of an active local RAAS within the human eye. It has been shown that at 12 h after a retinal ischemia-reperfusion injury, there is an upregulation of the protein levels of angiotensin II type 1 receptor (AT1-R) in the retina. Furthermore, at 12 h after reperfusion, there is an increase in reactive oxygen species (ROS) production in the retina that is mediated via an NADPH oxidase pathway. This ischemia-reperfusion injury-induced increase of retinal ROS levels and NADPH oxidase expression can be prevented by the administration of an AT1-R antagonist. This suggests that one of the main retinal ischemic injury pathways is via the local RAAS. It has also been reported that progressive retinal ganglion cell loss and glaucomatous optic nerve degeneration without elevated intraocular pressure occur after administration of local or systemic aldosterone. Elucidation of glaucoma pathogenesis, especially normal-tension glaucoma (NTG) subtype by our current animal model can be used for identifying potential therapeutic targets. Based on these results, we are further evaluating NTG prevalence among primary aldosteronism patients.

## 1. Introduction

Glaucomas are defined as optic neuropathy responsible for progressive degeneration of retinal ganglion cells (RGCs). More than 70 million people are affected by glaucoma worldwide, with approximately 10% classified as being bilaterally blind [1]. Thus, glaucomas are considered to be the leading cause of irreversible blindness in the world. Intraocular pressure (IOP) is one of the most important risk factors for the development and progression of glaucoma and is therefore a major target of the treatment of glaucoma. The upper limit of normal IOP is defined as 21 mmHg. Pooled data from the Ocular Hypertension Treatment Study (OHTS) and the European Glaucoma Prevention Study (EGPS) showed that for every 1 mmHg baseline IOP, there is a relative risk of 1.11 for glaucoma development [2]. IOP reduction is currently the only evidence-based treatment available for use in glaucoma patients [3]. In one report, glaucoma progression was detected in 45% of the treatment group, for a reduction of 25% that was maintained throughout the follow-up period, compared with 62% of the untreated group over five years [4]. When there is glaucomatous optic nerve head change in conjunction with glaucomatous visual field defects without elevated IOP, this is referred to as normal-tension glaucoma (NTG). Results of a North American and European long-term collaborative study have demonstrated that a 30% IOP reduction was able to positively affect the visual field loss progression in NTG [5]. Unfortunately, even when there is adequate IOP control, progression of glaucoma is sometimes observed. Moreover, it has been shown that NTG is a multifactorial disease, with progressive RGC death occurring even without an elevated IOP. Previous studies that have examined the fundamentals of glaucoma have reported finding associations with various systemic vascular diseases including low systemic blood pressure, transient nocturnal decreases in blood pressure, hypertension, migraine, vasospasm, and diabetes [6,7,8,9]. In addition, it has been suggested that impaired ocular blood flow, low intracranial pressure, apoptosis, autophagy, neurotrophins and excitotoxins, autoimmunity, and oxidative stress could potentially be non-IOP related risk factors associated with glaucoma progression [10].

One of the essential systems involved in the regulation of blood pressure is the renin-angiotensin-aldosterone system (RAAS). In the RAAS cascade, renin is involved in the first and rate-limiting step. Subsequently, it then binds to the liver-produced angiotensinogen to generate the decapeptide angiotensin I (Ang I). After hydrolysis by angiotensin converting enzyme (ACE) that is present in either the circulation or locally within the tissue, Ang I is converted to the oligopeptide angiotensin II (Ang II). After Ang II binds to the Ang II type 1 receptor (AT1-R), it then becomes the predominant physiological regulator of blood pressure. As such, when treating hypertension, this makes it one of the major targets of pharmacological intervention [11]. In addition, Ang II is involved in the release of aldosterone from the adrenal cortex. However, when Ang II binds to the AT2-R, this leads to the opposite effect of that observed after Ang II combines with the AT1-R, with vasodilation and decreasing fibrosis and inflammation subsequently observed. Furthermore, the chronic activation of the RAAS can lead to significant pathogenic actions by Ang II and aldosterone, which include cell proliferation, inflammation, oxidative stress and stimulation of fibrosis [12,13,14]. The effects of aldosterone, which is a steroid hormone, occur after its binding to the mineralocorticoid receptor (MR). The release of aldosterone is due to a variety of stimuli, which includes Ang II. Electrolyte and water balance in the body is associated with the binding of aldosterone to the MR, which has also been shown to influence the heart, kidney, and vascular pathology [15,16].

## 2. Targeting the Renin-Angiotensin-Aldosterone System to Treat Systemic Diseases

Generation of both Ang I and II is suppressed by renin inhibitors. For example, it has been demonstrated that agents such as aliskiren, which causes direct renin inhibition, can reduce blood pressure [17] and experimental atherosclerosis [18,19]. However, other studies have reported that aliskiren has protective effects against cardiovascular and renal injuries. There have also been extensive studies of cardiovascular and renal diseases that have examined traditional treatments that reduce the action of Ang II, including the use of an ACE inhibitor and AT1-R blocker (ARB) [20]. An increase in renal renin release that counteracts the effect of RAAS blockade has been shown to occur after ACE inhibitor and ARB treatments. Although it has been found that MR antagonists block the effects of aldosterone, they do not alter the Ang generation. A previous study of the kidney and heart reported enhanced MR signaling [21]. Furthermore, this study also evaluated eplerenone, which is an aldosterone antagonist, and reported that the progression of renal and cardiac diseases was dramatically delayed. Administration of either spironolactone [22] or an ACE inhibitor [23,24,25] in the stroke-prone spontaneously hypertensive rat, which is a genetic model of spontaneous hypertension, was reported to lead to large attenuations of both renal and cerebral vascular damage [25,26]. Administration of aldosterone in the remnant kidney hypertensive rat model has been similarly shown to lead to a reversal of the renal protection that occurs after the use of a combined ACE inhibition/ARB treatment to blockade the RAAS [27]. This phenomenon is referred to as the “aldosterone escape” [28], and it has been found that after these treatments, aldosterone may be present. As a result, independent of Ang II, this may be able to influence the pathology [29], with the activation of the AT1-R and ACE thereby potentiating the actions of Ang II [30,31]. Based on these findings, enhancement of the counter-regulatory arm of the RAAS is currently being evaluated as a possible treatment target for various diseases.

## 3. The Renin-Angiotensin-Aldosterone System in the Retina

Several organs, including the heart, adrenal gland, ovary, and thymus, have been shown to have a local RAAS [32,33,34,35]. The amounts of the RAAS components observed in both the ocular fluid and tissues in the eye suggest there is local production [36,37]. Both retinal pigmented epithelium (RPE) and retinal Müller cells express renin [38,39]. An evaluation of the plasma prorenin content of ocular fluid demonstrated that ocular prorenin was present at levels 100 times higher [40]. Thus, local production of these constituents is suggested. Furthermore, since evaluations of the anterior uveal tract, neural retina, RPE, and choroid of the normal porcine eye showed Ang I and Ang II were at levels 5- to 100-fold higher, respectively, which can be normally accounted for by blood contamination [26], this suggests that there are important local effects that are not associated with the circulating levels. In the rat, aldosterone synthase is expressed in Müller cells, retinal microvascular cells, and RGCs [41], whereas MR is found on vascular cells, RGCs, glia, and RPE [42,43]. The cleavage of Ang II by an ACE homolog, ACE2, produces Ang-(1-7), which also acts on the Ang II type 2 receptor and Mas receptor to partially antagonize the effects of AT1-R [44,45]. Mas receptor is expressed in different types of cells in the retina [46,47,48,49,50,51] and enhancing ACE2/Ang-(1-7) is protective in diabetic retinal neurovascular dysfunction and ocular inflammation [47,48,49,50,51]. In the human eye, Ang II and Ang-(1-7) are colocalized in retinal Müller cells and ACE2 is detected in the retina [52] In glaucoma, the primary cells affected are the RGCs. Regulation of blood flow [43] and IOP appears to be the main role of the local RAAS in the eye since this pressure can be lowered by renin and ACE inhibitors [53]. Studies of diabetic retinopathy treatments have also evaluated the use of ACE inhibitors and ARB. A 50% reduction in the progression of diabetic retinopathy and an 80% reduction in the progression to proliferative diabetic retinopathy were reported by the EURODIAB Controlled Trial of Lisinopril in Insulin-dependent Diabetes (EUCLID) after two years of treatment [54]. It is also possible that an increase in the progression of central serous chorioretinopathy (CSCR) and neovascular age-related macular degeneration (AMD) could potentially be caused by inappropriate activation of the MR, which leads to increased retinal fluid retention and dilation of choroidal vessels [55]. In chronic CSCR patients, an MR antagonist reduced subretinal fluid, subfoveal choroidal thickness, and visual acuity [56], whereas AMD patients exhibited decreased central retinal thickness, foveal thickness, and subretinal fluid [57].

The purpose of the present review was to specifically examine the role and potential mechanisms of aldosterone in the pathophysiology of glaucoma.

## 4. Association between the Renin-Angiotensin-Aldosterone System and Retinal Ischemia-Reperfusion Injury

A large number of retinal diseases such as glaucoma, diabetic retinopathy, and central retinal artery occlusion have been shown to be associated with retinal ischemia. Moreover, these diseases have been shown to be the leading cause of visual impairment or blindness [58,59,60]. There have been many mechanisms suggested as causes of tissue injury-induced ischemia [61,62,63]. For example, hypersecretion of glutamate and aspartate results from reactive oxygen species (ROS) triggering ischemic cell damage [64]. The ischemia-reperfusion that causes the production of an excess amount of glutamate then stimulates N-methyl-D-aspartate (NMDA), which is a subtype of the glutamate receptor [64], thereby inducing an excess Ca^2+^ influx into cells [61,62].

**Figure 1:**
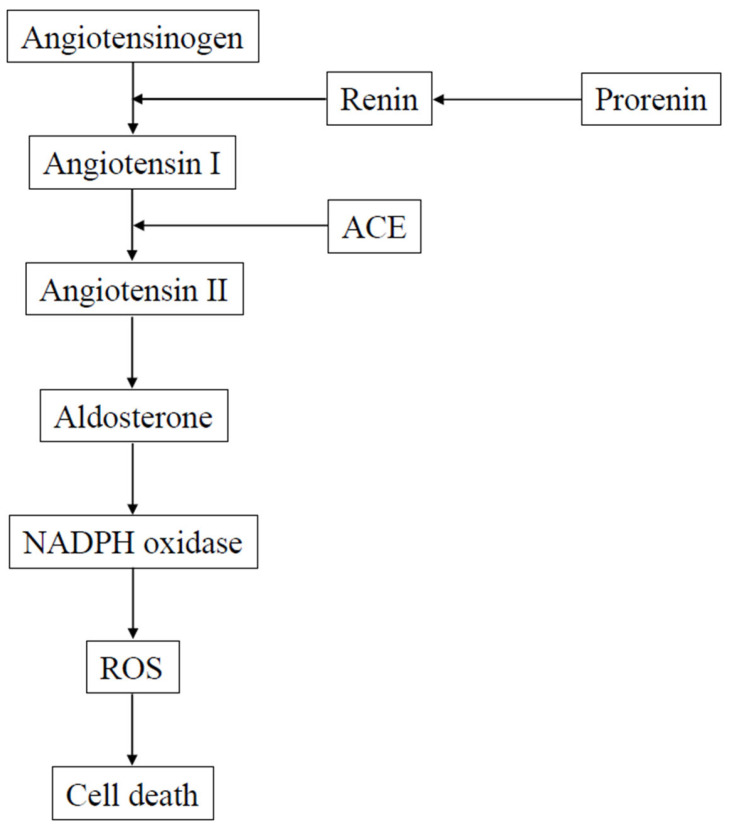
Possible mechanism of retinal neuronal cell death. After activation of the renin-angiotensin-aldosterone pathway, the production of reactive oxygen species in the retina is mediated via an NADPH oxidase pathway. ACE, angiotensin converting enzyme; ROS, reactive oxygen species.

It has been shown that the inner retina, including RGCs, is damaged seven days after retinal ischemia-reperfusion injury [65]. Release of glutamate from the rat retina was observed during the ischemic period, and a large increase was observed during reperfusion [66]. In ischemia-reperfusion, neurotransmitters overactivate their appropriate receptors. Such overstimulation, particularly of ionotropic glutamate receptors, generally leads to cell death. RGC survival rates at seven days after retinal ischemia-reperfusion injury were 46–58% [65,67,68]. However, prevention of retinal ischemia-reperfusion injury can be achieved by administering a direct renin inhibitor [67], ACE inhibitor [65], AT1-R antagonist [65,69], or MR antagonist [68]. Blocking the RAAS at its point of origin, the renin-angiotensinogen interaction can be achieved by the use of direct renin inhibitors, which are antihypertensive drugs [70]. As a result, conversion of angiotensinogen to Ang I is prevented, which leads to the interruption of the RAAS cascade. The formation of Ang II from Ang I can be prevented by ACE inhibitors. This then reduces the action of Ang II at both the AT1-R and AT2-R. However, it should be noted that AT1-R antagonists have been shown to act more selectively by blocking the action of Ang II at the AT1-R. It has been shown that at 12 h after reperfusion, the protein levels of AT1-R are upregulated in the retina [65]. In addition, at 12 h after reperfusion, an increased ROS production is observed in the retina, with the increased levels associated with an increase in the p47phox and p67phox mRNA expressions [69]. These findings suggest that an NADPH oxidase pathway at 12 h after reperfusion is responsible for mediating the ROS production in the retina. Another study has shown that administration of the AT1-R antagonist, candesartan, was able to prevent the ischemia-reperfusion injury-induced increase of both retinal ROS levels and NADPH oxidase expression [69]. Thus, these results demonstrated that one of the main pathways of retinal ischemic injury is via the local RAAS. A complete reversal of the RAAS suppression-induced neuroprotective effect against the retinal ischemia-reperfusion injury was also observed after the administration of aldosterone in rats receiving an AT1-R antagonist [68]. Moreover, a neuroprotective effect against retinal ischemia-reperfusion injury was observed with the MR antagonist [67]. When taken together, these findings demonstrate that ischemic damage in the retina can be influenced by MR and aldosterone. Figure 1 presents the details on a possible mechanism associated with the retinal neuronal cell death mediated by the local RAAS.

## 5. Aldosterone and Retinal Ganglion Cells

A significant decrease in the number of RGCs in the normal rat was observed even though an intravitreal injection of aldosterone without ischemia did not affect the retinal thickness [68]. It has been suggested that one potential risk factor for RGC death is associated with glutamate excitotoxicity triggered by the overactivation of the NMDA receptors [70]. Results of an animal model demonstrated that after intravitreal injection of NMDA there is a decrease in the RGCs [71]. These findings suggest that it is important that the relationship between the RAAS and the NMDA receptor-mediated signal and the prevention of RGC death be further investigated. Although there was protection against RGC death after intravitreal injection of aldosterone by the MR antagonist, spironolactone, there was no protection observed after the administration of the non-competitive NMDA receptor antagonist, memantine [72]. However, after intravitreal injection of NMDA, memantine but not spironolactone protected against RGC death [72]. Therefore, these findings indirectly indicate that downstream of the NMDA receptor-mediated signal, the RAAS does not exist, and in addition, that downstream of the RAAS the NMDA receptor-mediated signal does not exist. Moreover, both the RAAS and NMDA receptor-mediated signals appear to be important pathways that are associated with RGC death. As a result, both the NMDA receptor-mediated signal and the RAAS need to be taken into careful consideration when evaluating the use of neurotherapeutics for glaucoma management.

Hematoxylin and eosin-stained retinal sections were used to examine the morphology of each retinal layer [73]. The thickness of the inner plexiform layer, inner nuclear layer, outer plexiform layer, or outer nuclear layer appeared to be unaffected after systemic administration of aldosterone (80 μg/kg/day) [73]. Although there was no effect on other retinal neurons, progressive RGC loss, and glaucomatous optic nerve degeneration without elevated IOP were observed at six weeks after systemic administration of aldosterone [73]. Therefore, this NTG rat model appears to be effective for investigating mechanisms of neurodegeneration in NTG in addition to assisting in the development of therapies that can be directed at IOP-independent mechanisms of RGC loss. Furthermore, in this animal model, it has been shown that spironolactone administration can prevent RGC loss [73]. Other studies have reported that apoptosis of proximal tubular cells [74], mesangial cells [75], and cardiac myocytes [76] is induced by aldosterone in a ROS-dependent manner. When these findings are taken together with those of our previous studies [65,68,69], this model appears to suggest that aldosterone induces RGC death in a ROS-dependent manner. Examination of the ganglion cell layer showed the presence of TUNEL-positive cells after systemic administration of aldosterone [77]. Therefore, this indicates that cell death from apoptosis is responsible for the loss of RGCs. In a previous monkey model of pressure-induced glaucoma, it was reported that apoptosis was at least one of the mechanisms responsible for RGC death [78]. However, the definitive cell death mechanism in this particular animal model remains unknown. Therefore, gene expression changes in the retina after systemic administration of aldosterone will need to be investigated. An analysis of the microarray data sets after systemic administration of aldosterone demonstrated there was an upregulation of 24 genes and downregulation of 24 genes of the key apoptosis-specific genes [79]. Furthermore, after performing real-time PCR, the results demonstrated that 5 genes (*Bcl3*, *Cdkn1a*, *Tbox5*, *PF4*, and *Vdr*) were upregulated while 10 genes (*Asns*, *Bard1*, *Card9*, *Fcgr1a*, *Inhba*, *Kcnh8*, *Lck*, *Phlda1*, *Ptprc*, and *Sh3rf1*) were downregulated [79]. This suggests that after systemic administration of aldosterone, it could be possible that there are two mechanisms associated with the RGC death. First, the death of the RGCs might be associated with ocular blood abnormalities due to the upregulation of PF4. Second, increases of the ROS levels could lead to the induction of p53 activation as an upstream signal, with a net result of the triggering of apoptosis. Uncontrolled production of ROS is recognized to be an important mechanism of apoptosis in neurodegenerative diseases, including glaucoma [80]. The axons of RGCs contain a large number of mitochondria; they have a greater energy demand and are more sensitive to ROS stress [81]. To definitively clarify the mechanisms of the loss of RGCs and their axons after systemic administration of aldosterone, further investigations will need to be conducted.

After systemic administration of aldosterone, an increase in plasma aldosterone concentrations was observed [77]. Moreover, an evaluation of the relationship between the plasma aldosterone concentration and the number of RGCs showed a negative correlation [77]. Subsequent study evidence suggested that aldosterone was independently involved with cardiovascular injury in the kidney and brain [82]. Thus, plasma aldosterone concentration determinations are of importance in helping to prevent organ complications, including in the retina. Based on these findings, it is necessary to carefully take plasma aldosterone concentrations into consideration.

## 6. Aldosterone and Blood Flow

In the vascular endothelium, aldosterone is known to cause changes via acute, non-genomic, and chronic, genomic effects that subsequently modulate vascular resistance and blood flow. High plasma aldosterone concentrations have been shown to be responsible for vasculopathy [83]. Another study has shown that reduction of endothelial nitric oxide (NO) synthesis and bioavailability along with the increased generation of superoxide radicals that degrade endogenous NO are characteristics of aldosterone-induce vasculopathy [82]. Moreover, activation of MRs in the rat kidney and colon is associated with aldosterone-induced gene expression of endothelin 1 (ET-1), which is a vasoconstrictor peptide synthesized by vascular endothelial cells [84]. In isolated porcine retinal veins, ET-1 was shown to induce dose-dependent vasoconstriction [85]. Furthermore, aldosterone in experimental models and humans has been shown to cause blood flow changes. Fujita et al. [86] examined an acute aldosterone infusion into the left anterior descending coronary artery in open-chest dogs and found there was a decreased coronary blood flow. In humans, Romagni et al. [87] reported that after an aldosterone infusion into the antecubital vein of the arm, there was a rapidly decreased forearm blood flow effect. In addition, there was a decrease in optic nerve head (ONH) blood flow following retinal vessel constriction without changes in IOP or systemic blood pressure after systemic administration of aldosterone in rats [88]. Since there was stable blood pressure and pulse rate during the experimental periods, this reduction in the ONH blood flow following vessel constriction most likely reflects the local effects of aldosterone on the rat vessels in the ONH.

## 7. Primary Aldosteronism and Normal-Tension Glaucoma

Plasma aldosterone concentrations ranged from 368 to 527 pg/mL after systemic administration of aldosterone at a dose of 80 μg/kg/day [77]. To definitively identify suspected primary aldosteronism (PA), this required that the plasma aldosterone concentrations had to be greater than 150 pg/mL [89]. Within the setting of a low plasma renin, PA is defined as an inappropriate elevated aldosterone. Previously, PA was considered to be a rare and niche secondary cause of hypertension. However, as it has been shown there is a prevalence of approximately 20% among patients with resistant hypertension [90,91], 10% in patients with severe hypertension (systolic blood pressure ≥180 mmHg, diastolic blood pressure ≥110 mmHg) [92,93], and 6% in patients with otherwise uncomplicated hypertension, PA is now considered to be the most common cause of secondary hypertension [93]. However, only a small fraction of the PA patients can be diagnosed and treated, even though PA is the most frequent cause of secondary hypertension [93]. When PA is compared with primary hypertension, it has been shown that there is a higher risk for coronary artery diseases [94,95,96,97,98], atrial fibrillation by itself or in the context of other heart diseases [94,95,96,97,98], stroke [80,81,82,83,84], left ventricular hypertrophy and/or heart failure [94,96], metabolic syndrome and/or diabetes mellitus [94], kidney diseases [99,100], and decreased bone density and fracture [101]. The effect of MR activation by aldosterone within the volume expansion setting is most likely the reason for this excess risk. Although population-based studies have reported finding an association between hypertension and glaucoma [8,102,103], the exact relationship between NTG and PA remains unknown. Moreover, the association between incident glaucoma and systolic or diastolic blood pressure has yet to be verified by other prospective studies [104,105]. It is possible that there were fewer patients with hypertension due to PA when these studies were compared, and this may be the reason for this discrepancy. The blood-retinal barrier is known to maintain homeostasis in the retina. So far, we do not know the blood-retinal barrier penetration of aldosterone. After systemic administration of aldosterone, however, decreased ONH blood flow [88], decreased number of RGCs [73], and changed gene expression in the retina [79] have been observed. These results indirectly showed that an effective concentration of aldosterone did reach the retina. Additional studies that investigate the prevalence of NTG among patients with PA will need to be conducted in the future.

## 8. Conclusions

One of the main pathways of retinal neuronal injury is the RAAS, which exists within the retina. There is increasing evidence that aldosterone may play a role in eye diseases. There is considerable evidence that the RAAS plays a role in diabetic retinopathy, retinal vein occlusion, age-related macular degeneration, and retinopathy of prematurity [104,105]. MR inhibition could be a therapeutic target in these diseases [106,107]. Further studies focusing on aldosterone-mediated effects on retinal diseases are needed.

Glaucoma is a very complex disease and is known to lead to irreversible blindness in many people. The loss of RGCs and their axons in the rat after systemic administration of aldosterone was shown to be a time-dependent loss without elevated IOP. This current animal model appears to be an effective tool that can be used to investigate NTG neurodegeneration mechanisms in addition to being used to help develop therapies directed at the IOP-independent mechanism of RGC loss. One of the risk factors for developing glaucoma appears to be increased plasma aldosterone levels. Therefore, the relationship between NTG and PA will need to be clarified by further definitive clinical studies. At the present time, additional studies designed to investigate the prevalence of NTG among patients with PA are currently being conducted by our research group.
